# Transoral Endoscopic Thyroidectomy Vestibular Approach (TOETVA): Surgical Outcomes and Learning Curve

**DOI:** 10.3390/jcm10040863

**Published:** 2021-02-19

**Authors:** Young Jun Chai, Sumin Chae, Moon Young Oh, Hyungju Kwon, Won Seo Park

**Affiliations:** 1 Department of Surgery, Seoul National University Boramae Medical Center, Seoul 07061, Korea; kevinjoon@naver.com; 2 Department of Surgery, Kyung Hee University College of Medicine, Kyung Hee University Medical Center 23, Kyungheedae-ro, Dongdaemun-gu, Seoul 02447, Korea; oparms1@gmail.com; 3 Department of Surgery, Seoul National University Hospital, Seoul 03080, Korea; oh.moon.young@gmail.com; 4 Department of Surgery, Ewha Womans University Medical Center, Seoul 07985, Korea; lovekkung@gmail.com

**Keywords:** transoral endoscopic thyroidectomy vestibular approach, minimally invasive surgery, thyroid

## Abstract

The transoral endoscopic thyroidectomy vestibular approach (TOETVA) has excellent cosmetic effects and its popularity is increasing worldwide. We present our experience with TOETVA and its short-term outcomes. This study included 110 consecutive patients who underwent TOETVA at a single institution between July 2016 and June 2020. We analyzed clinicopathologic data, short-term postoperative outcomes, and learning curve using cumulative summation (CUSUM) analysis. Of the 110 patients who underwent TOETVA, 101 had malignant disease and 100 (90.9%) underwent lobectomy. The mean age was 39.7 ± 9.7 years, and the mean tumor size was 1.0 ± 0.7 cm (range, 0.3–3.6 cm). Operation time was 168.0 ± 63.4 min for total thyroidectomy, 111.0 ± 27.7 min for lobectomy, and 73.7 ± 18.1 min for isthmusectomy. Five patients (4.5%) experienced transient vocal cord palsy (VCP) and one (0.9%) had permanent VCP. The swallowing impairment index-6 score was 2.18 ± 3.21 at postoperative three months, and 0.97 ± 1.72 at postoperative six months. The learning curve for lobectomy was 58 cases in CUSUM analysis. TOETVA is a safe and feasible approach with an acceptable operation time and a low complication rate. This approach is a surgical option for patients who desire excellent cosmesis.

## 1. Introduction

Open thyroidectomy using the traditional Kocher incision remains the main approach to treat thyroid nodules. However, this method produces a scar on the anterior neck resulting in poor cosmetic outcomes. Since the first endoscopic parathyroidectomy was performed in 1996 [1], minimally invasive endoscopic or robotic approaches have been developed and modified to minimize visible scarring and produce better cosmetic results. These remote access approaches have become popular and present excellent alternative options to conventional thyroid surgery by moving the scar from the anterior neck to other parts of the body, such as the axilla, breast, and retro-auricular area. However, while these approaches make the scars unnoticeable, cutaneous scarring is not invisible. Moreover, they increase operation time and lead to greater postoperative pain due to extensive flap dissection to reach the thyroid. Thus, they cannot be classified as truly minimally invasive surgery.

The transoral endoscopic thyroidectomy has two approaches: sublingual and vestibular. A transoral endoscopic thyroidectomy vestibular approach (TOETVA) was first described in 2016 [2], and several studies report encouraging results with few complications [3,4]. Due to its advantages, including complete healing without scar, reduced operation time and range of dissection, and a short learning curve, TOETVA has been reported to be an ideal minimally invasive thyroidectomy technique [5,6]. However, few large-scale studies have been conducted, especially for malignant tumors [7,8]. Here, we report a TOETVA case series, including 101 malignant cases performed by a single surgeon, and demonstrate the procedure’s safety and feasibility.

## 2. Materials and Methods

### 2.1. Patients

We collected medical record data from all patients who underwent TOETVA at the Seoul Metropolitan Government Seoul National University Boramae Medical Center from July 2016 to June 2020. Indications for TOETVA were nodules <4 cm, without lateral neck node metastasis or extensive central node metastasis on preoperative ultrasonography. Contraindications were (1) prior neck surgery and irradiation, (2) oral abscesses, and (3) substernal goiter. Prior chin surgery was considered a relative contraindication, and inclusion of such patients was determined on a case-by-case basis. After a thorough explanation of the advantages and disadvantages of TOETVA, including TOETVA-specific complications such as chin numbness, subcutaneous emphysema, and CO_2_ embolism, the patients themselves decided whether to undergo TOETVA or other approaches. In our institution, TOETVA was first performed in July 2016, and the first 110 operations performed by a single surgeon were included in this study. This study was approved by the institutional review board of the Seoul National University Boramae Medical Center (IRB No. BTMP-2020-191) and written informed consent to participate in this study was waived.

### 2.2. Surgical Procedures

**Figure 1:**
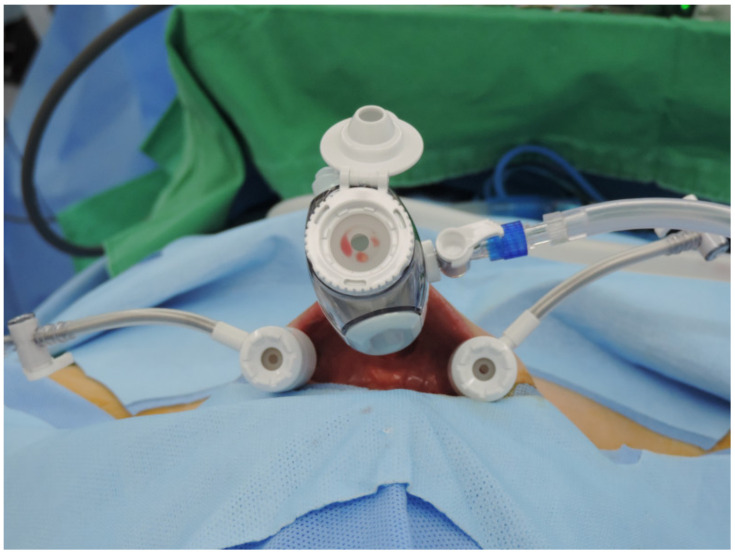
Trocar placement for transoral endoscopic thyroidectomy vestibular approach.

All procedures were performed by a single experienced surgeon (Y.J. Chai). An electromyographic endotracheal tube (Medtronic, Jacksonville, FL, USA) or adhesive electrodes (Inomed, Emmendingen, Germany) were used for intraoperative neuromonitoring (IONM). Prophylactic antibiotics were administered 30 min before surgery (Cefotetan, 1 g). Patients were placed in a supine position with the neck extended slightly, as in conventional thyroid surgery. After positioning, the oral cavity was washed with a chlorhexidine solution to prevent infection. Three linear incisions (midline, 2 cm; bilateral, 0.5 cm) were made in the lower oral vestibule. For better mobility, the midline incision was made 1 cm above the frenulum of the lower lip. Hydrodissection was performed with 30 mL of epinephrine–saline diluted solution (1:200,000) administered via the incisions down to the subplatysmal plane. Blunt tunneling using an 8 mm-tipped vascular surgical tunneler created the working space. A 10-mm trocar was inserted through the midline incision, and CO_2_ insufflation was maintained at 5 mmHg with a high flow rate. Bilateral incisions were made at 1 cm medial to both labial commissure of the mouth. Two 5-mm trocars were inserted through the lateral incisions into the working space. Trocar placement is shown in Figure 1. A 10-mm 30° 4K scope was inserted through the midline trocar with craniocaudal visualization. Grasper forceps, L-hook cautery, and ultrasonic shears were inserted through the lateral trocars.

**Figure 2:**
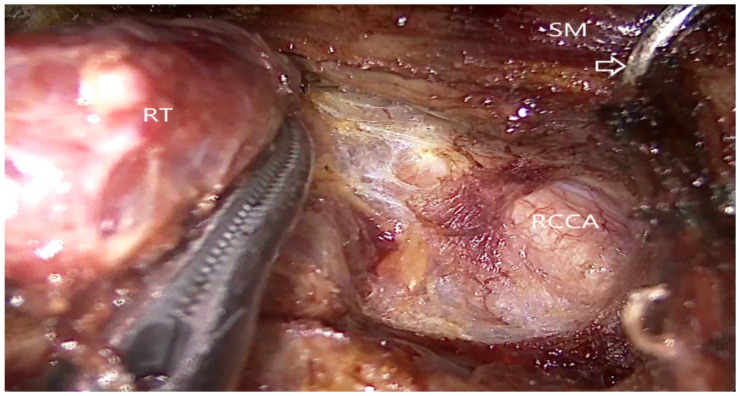
Endoscopic view of the transoral endoscopic thyroidectomy vestibular approach. RT: right thyroid gland, RCCA: right common carotid artery, SM: strap muscle, Arrow: external retractor.

After further flap dissection to widen the working space, the midline of the sternohyoid muscle was divided using an L-hook cautery. The isthmus was transected using the ultrasonic shears, and the thyroid lobe was medially retracted. The lateral aspect of the thyroid was dissected by separating the thyroid from the sternothyroid muscle, and the strap muscles were retracted using an external retractor (Figure 2).

**Figure 3:**
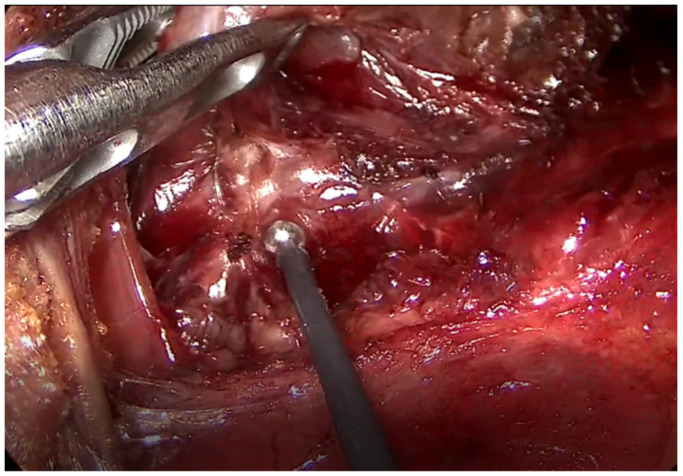
External branch of the superior laryngeal nerve monitoring using a long ball-tip monopolar stimulation probe.

The superior pole was lifted and dissected along an avascular plane, and the superior thyroidal vessels were divided and ligated using the ultrasonic device. To preserve the external branch of the superior laryngeal nerve (EBSLN), nerve stimulation was performed along its course before and after sealing the superior thyroidal vessels using a neural integrity monitor (NIM-Response 3.0 System; Medtronic, Jacksonville, FL, USA). The functional integrity of the EBSLN was monitored using a long ball-tip monopolar stimulation probe (Figure 3).

**Figure 4:**
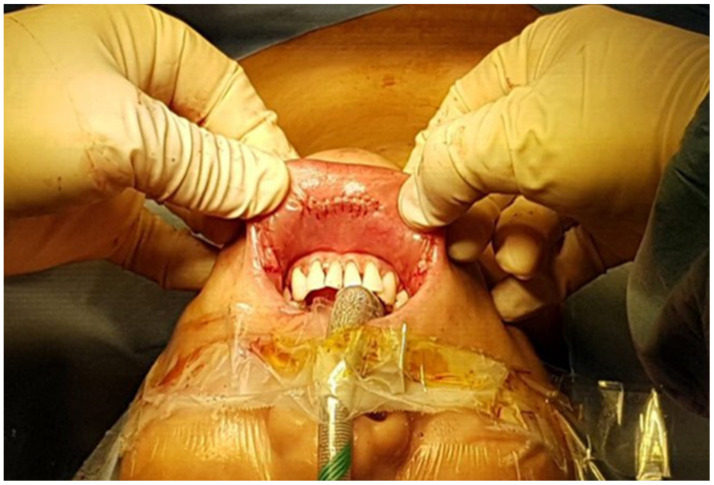
Oral mucosal incisions after suturing.

After dissecting the adjacent soft tissue, the superior parathyroid gland was exposed and preserved. The thyroid gland was meticulously dissected, and the recurrent laryngeal nerve (RLN) was identified at its entry point near the Berry’s ligament. While dissecting in the craniocaudal direction, the thyroid was resected from the trachea after preservation of the inferior parathyroid gland. Prophylactic or therapeutic central lymph node dissection (CLND) was routinely performed in patients with a malignant nodule diagnosed by preoperative fine needle aspiration. The specimen was removed using an endopouch through the midline incision. When the specimen was too large for the midline incision, 13 to 18 mm Hegar cervical dilators were used in a stepwise manner to widen the incision. The oral mucosal incisions were closed using 4-0 absorbable sutures (Figure 4). To prevent postoperative bleeding, a compression dressing was applied to the chin and anterior neck area.

### 2.3. Surgical Outcome Measurement

The following surgical outcomes were collected by medical chart review: extent of surgery, operation time, pathologic results, length of hospital stay, open conversions, and postoperative complications. Operation time was measured from oral vestibular incision to closure. Tumor size was determined by the longest diameter of the tumor. For malignant tumors, T and N stages were classified by the AJCC 8th edition staging system. Length of hospital stay was measured from the day of surgery to the day of discharge. Vocal cord palsy (VCP) was defined as loss of vocal cord mobility. Vocal cord mobility was routinely assessed with a 70° rigid laryngoscope preoperatively and at the first visit after discharge. In cases of VCP, laryngoscopic examinations were performed regularly until vocal cord mobility was restored. Permanent VCP was determined as vocal cord immobility that persisted longer than six months. Occurrence of hypoparathyroidism was investigated in patients who underwent total thyroidectomy. Hypoparathyroidism was defined as serum parathyroid hormone (PTH) level <15 pg/mL, requiring oral calcium supplementation. Hypoparathyroidism that persisted less than six months was defined as transient hypoparathyroidism. Swallowing Impairment Index-6 (SIS-6) scores were used to assess swallowing outcomes [9]. The SIS-6 is a self-evaluation questionnaire consisting of six items about swallowing dysfunction and symptom such as dysphagia, cough, choking, and throat clearing. The total score ranges from 0 to 24, and higher score indicates more severe impairment. Questionnaires were conducted at postoperative three and six months.

### 2.4. Statistical Analysis

The chi-square test was used to analyze the frequency differences between groups for categorical data, and the Wilcoxon rank sum test and Kruskal–Wallis test were used for continuous data. The Bonferroni correction was used for post-hoc analysis. Multiple logistic regression was performed to determine the association, and the Firth penalized likelihood approach was used to reduce bias for small sample size. Cumulative summation (CUSUM) analysis was used to analyze the learning curve. The slope of the CUSUM curve represents the trend of learning outcomes, and the point at which the slope changes from positive to negative is regarded as the point of overcoming the learning curve. We rejected null hypotheses of no difference if p-values were less than 0.05. Statistical analysis was performed using the SAS 9.4 software (SAS Institute Inc., Cary, NC, USA.) and the “qcc” package of R 4.0.2 (R Foundation for Statistical Computing, Vienna, Austria. URL http://www.R-project.org, accessed on 9 November 2020).

## 3. Results

### 3.1. Demographics and Surgical Outcomes

A total of 110 patients (9 males, 101 females) were included in the analysis. Patient demographics and pathological outcomes are listed in Table 1. The mean age was 39.7 ± 9.7 years, and the mean body mass index (BMI) was 23.5 ± 4.4 kg/m^2^. The mean tumor size was 1.0 ± 0.7 cm (range 0.3–3.6 cm), and 101 patients underwent TOETVA due to malignant disease. Table 2 displays operative outcomes and surgical complications. The mean operation time was 111.0 ± 27.7 min for lobectomy, and 168.0 ± 63.4 min for total thyroidectomy. The number of retrieved lymph nodes was 4.1 ± 3.5. Postoperative VCP occurred in six patients (5.4%), five of whom recovered within six months. Transient hypoparathyroidism was observed in one of the three patients who underwent total thyroidectomy. The average length of postoperative hospital stay was 2.4 ± 0.9 days. The median follow-up period was 15.6 (range, 3.3–51.9) months.

There was one case of postoperative bleeding in a patient (40-year-old female) who underwent lobectomy due to papillary thyroid carcinoma (PTC). One hour after the operation, postoperative bleeding was suspected because of lower lip swelling. Repeat endoscopic surgery was performed, and bleeding at the mandible area was identified and successfully controlled. No other complications, such as seroma collection, surgical site infection, or mental nerve injury, occurred.

Two patients had open conversion. In the first case (33-year-old female), a 1.4 cm-sized tumor was visualized in the Berry’s ligament by preoperative ultrasonography. Conventional open thyroidectomy was recommended under suspicion of RLN invasion. However, TOETVA was first attempted at the request of the patient. After confirming RLN invasion of the tumor, the approach was converted to open surgery and total thyroidectomy was performed. In the second case (43-year-old female), there was bleeding from the anterior jugular vein during flap dissection, and end tidal CO_2_ increased to 50 mmHg immediately, and systolic blood pressure dropped to 60 mmHg about one minute later. Under the suspicion of CO_2_ embolism, CO_2_ insufflation was halted, and blood pressure recovered immediately. The approach was converted to open lobectomy, and the patient had no further complications related to CO_2_ embolism or bleeding.

Two patients underwent additional thyroidectomy. In one case, the patient (35-year-old female) initially underwent isthmusectomy due to a 4 mm PTC. Twelve months later, a 3 mm nodule in the left lobe was found on routine ultrasound. Fine needle aspiration was performed at the patient’s request. Results indicated suspected PTC, and open left lobectomy was performed. No further recurrence was observed after the second operation during the 30-month follow-up period. In the second case, the patient (36-year-old male) had previously undergone lobectomy, and occult lymph node metastases were found in all six retrieved lymph nodes, and extensive lymphatic invasion was evident on pathology. The patient underwent open thyroidectomy and radioactive iodine treatment. There was no recurrence during the following 14 months.

The mean operation time of lobectomy for malignant nodule was 112.1 ± 28.4 min. Patients were divided into a short-time group (>112 min) and a long-time group (≤112 min) based on the mean operation time. The mean tumor size of the long-time group was larger than that of the short-time group (1.2 ± 0.8 vs. 0.8 ± 0.4, *p*-value, 0.012).

Age, BMI, laterality, LN metastasis, extrathyroidal extension (ETE), and thyroiditis were equally distributed between groups (Table 3). In multivariable analysis, tumor size was a significant independent factor affecting the operation time (OR, 2.53; 95%CI, 1.05–6.09; *p*-value, 0.039) (Table 4). The operation times were 111.2 ± 29.5 min for BMI <25, and 115.0 ± 25.1 min for BMI ≥25. There were no complications in patients with BMI ≥25. Sixty patients completed the SIS-6 questionnaires for assessment of swallowing impairment at postoperative three and six months. The score at postoperative three months was 2.18 ± 3.21, and thyroiditis and tumor size were significant independent factors affecting SIS-6 in multivariable analysis (β estimate, 2.69; 95% CI, 0.56–4.82; *p*-value, 0.015 and β estimate, 1.63; 95% CI, 0.35–2.91; *p*-value, 0.015, respectively). At postoperative six months, the score of SIS-6 decreased to 0.97 ± 1.72.

### 3.2. CUSUM Analysis for Learning Curve

**Figure 5:**
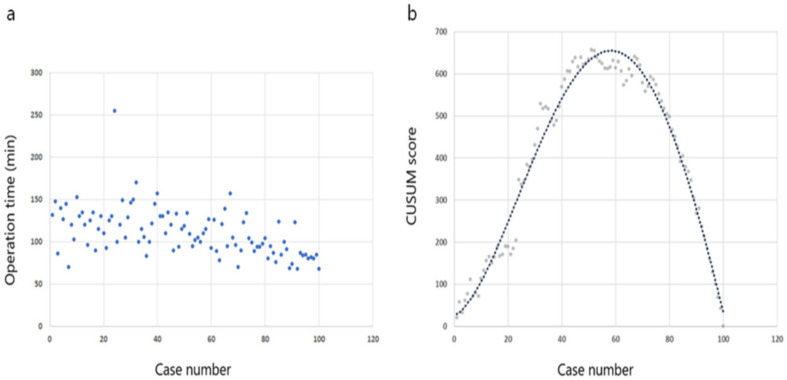
Operation time of lobectomy with transoral endoscopic thyroidectomy vestibular approach. (**a**) Operation time plotted in chronological order. (**b**) Cumulative summation test of operation time.

CUSUM analysis was performed based on chronological cases to evaluate the learning curve in unilateral lobectomy cases. The CUSUM curve and operation times are shown in Figure 5. The best fit for the curve was a fourth-order polynomial with equation CUSUM equal to 25.232 + 3.457 × (Case number) + 0.564 × (Case number)^2^ − 9.656 × 10^−3^ × (Case number)^3^ + 3.678 × 10^−5^ × (Case number)^4^, which had a high *R*^2^ value of 0.975. The operation time CUSUM chart showed a curve with a positive slope from the initial case to case 57. After case 58, the CUSUM curve showed negative slope periods, indicating that the learning curve for TOETVA was 58 cases. Therefore, the curve was divided into two phases: phase 1, the learning period (cases 1–57) and phase 2, the proficient period (cases 58–100). The operation time in phase 2 was significantly shorter than that in phase 1 (96.8 ± 20.9 vs. 121.8 ± 27.6; *p*-value, <0.001). Postoperative VCP was observed in four cases (7.0%) in phase 1, and two cases (4.7%) in phase 2 (*p*-value, 0.697).

## 4. Discussion

TOETVA has become attractive to thyroid surgeons and patients around the world because it addresses the desire for minimally invasive surgery and enhanced aesthetic outcomes. Numerous studies have published encouraging results [3,5,6,10]. In our study, the operative time for lobectomy was comparable to other reports of initial TOETVA experience [11,12]. Furthermore, the TOETVA complication rates in this study were in line with acceptable rates following open thyroidectomy [13].

Surgery on obese patients is considered technically challenging, time consuming, and is associated with higher complication rates. Particularly in transoral thyroid surgery, the heavy subcutaneous layer can increase tension over the skin flap and lead to fat necrosis, wound infection, and seroma formation [14]. Studies report that obesity does not significantly affect TOETVA outcomes such as operation time or postoperative complications [15,16]. TOETVA has been successfully performed in patients with BMI >40 [17]. Compared to other remote access thyroidectomy approaches, the short access distance of TOETVA may be an advantage for obese patients. In the current study, obesity was determined based on a BMI ≥25 in accordance with the WHO guidelines for the Asia–Pacific region [18]. This study included 22 obese patients, and the procedure was safely performed in all 22 obese patients without delayed operation time or complications.

Although an endoscopic approach provides an excellent magnified view of the RLN [19], there is concern about the increased risk of RLN injury during TOETVA compared to open thyroidectomy. The mechanisms for RLN injury in TOETVA include (a) surgeon’s unfamiliarity with the cranio-caudal view and endoscopic instruments; (b) limited landmarks for RLN identification and modalities for nerve dissection; and (c) insufficient countertraction [20]. However, several studies found the overall incidence of RLN injury of 3.1–5.9% for TOETVA to be comparable to the incidence of 2.1–11.8% for open thyroidectomy [16,21,22,23]. The low incidence of RLN injury in TOETVA can be explained by the reduced incidence of traction injury and the use of IONM [23,24]. During TOETVA, the RLN is usually identified at its insertion site and released first after division of the Berry’s ligament. This reduces the risk of traction injury. In the current study, IONM was used in all cases and VCP occurred in six (5.4%) patients, five of whom recovered completely within six months of surgery. This is comparable to the literature [16,21].

The mental nerve (MN) is a branch of the posterior trunk of the inferior alveolar nerve originating from the mandibular nerve. One of the major concerns in TOETVA is MN injury, which causes numbness and paresthesia in the chin or lower lip, impairing quality of life [25]. Possible causes of MN injury include (a) incorrect vestibular incision and dissection; (b) imprecise port insertion and removal; (c) over-stretching and longer compression of trocars on the MN; and (d) anatomic variations of MN [26]. To protect the MN, surgeons should have a detailed anatomical knowledge of the MN, including its possible variations, and manipulation should be carefully performed during the operation [26]. Others observed MN injury in 1 to 5% of TOETVA cases [27,28,29]. In the current study, there was no MN injury. To prevent MN injury, vestibular incision and port insertion methods were standardized for accuracy and precision, and tension on the MN was minimized by avoiding over-stretching of the lateral two ports.

Surgical site infection (SSI) is another concern of TOETVA. While conventional thyroidectomy is considered a clean procedure, TOETVA is classified as a clean–contaminated procedure because the oral cavity is colonized by bacterial flora, Gram-positive aerobes, and anaerobes. Therefore, TOETVA warrants antibiotic prophylaxis [27]. SSI can develop into abscesses, and systemic infection such as sepsis can develop in severe cases. Reports show very low infection rates following a regimen of perioperative intravenous antibiotics (amoxicillin–clavulanic acid 1.2 g or oxacephem 1 g) for 1–2 days, followed by oral antibiotics for 3–7 days post surgery [3,16,30,31]. In the current study, to prevent SSI, preoperative antibiotic prophylaxis (Cefotetan 1 g) was administered once, and the oral cavity was carefully prepared. Even without additional antibiotics, no SSI occurred. We suggest that SSI can be prevented with preoperative antibiotics and careful oral cavity preparation in patients without gingival abscess. Further studies are warranted to determine the appropriate dosage and duration of antibiotics.

Postoperative bleeding is a severe complication of thyroid surgery that can lead to serious consequences, including airway obstruction. The risk of postoperative bleeding may be reduced by appropriate patient selection and careful surgical manipulation. In a systematic review of transoral thyroidectomy, the incidence of hematoma requiring subsequent surgery was extremely low (0.1%) [16]. Importantly, bleeding from the flap may be hidden during the operation, due to CO_2_ pressure, and only discovered after surgery. In this study, there was one case of immediate reoperation due to postoperative bleeding; oozing blood was found at the mandible flap. In all subsequent cases, a compression dressing with elastic plasters was applied on the chin and jaw to prevent postoperative bleeding. This is in accordance with several published recommendations that a compression dressing be applied to the chin and anterior neck for 12–24 h post surgery [3,16,30]. Ultrasonic shears were used for ligation of thyroid vessels in all cases. The use of ultrasonic shears provides safe and effective control of thyroid vessels and considerably reduces the risk of postoperative bleeding [32].

While seroma is a minor complication in endoscopic thyroidectomy, a large dissection area increases the risk of seroma collection. The use of drains is still controversial in thyroid surgery [33]. In endoscopic surgery, the risk of seroma collection may be reduced by image magnification and the use of ultrasonic shears. In this study, drains were not used in any cases, and there was no incidence of seroma collection.

To maintain the working space during endoscopic surgery, CO_2_ is insufflated. While CO_2_ embolism is quite rare, such occurrence can be fatal. The common cause of CO_2_ embolism during endoscopic thyroidectomy is the inadvertent injection of CO_2_ into an injured vessel during flap dissection [34]. Symptoms of CO_2_ embolism vary from asymptomatic to life-threatening, depending on the amount of CO_2_ entrapped. Thus, rapid diagnosis and immediate action are imperative. The gold standard for detecting CO_2_ embolism is transesophageal echocardiography. However, end tidal CO_2_ (EtCO_2_) monitoring is most convenient and effective for immediate diagnosis in the operating room [35]. Due to the increased pressure caused by the limited working space in TOETVA, a low insufflation pressure is essential to prevent intraoperative CO_2_ embolism [36]. Therefore, CO_2_ insufflation pressure was maintained at less than 5 mmHg during the operation in all cases. In previous studies, the incidence of CO_2_ embolism was 0.6–2.5% [37,38]. In this study, there was one case of suspected CO_2_ embolism during flap dissection. Insufflation of CO_2_ was immediately halted, and the patient’s vital signs spontaneously recovered without need for resuscitation. After open conversion, thyroidectomy was completed without any further complication. As protocols for preventing CO_2_ embolism are not yet clearly established, the surgeon and anesthesiologist should be aware of procedures for the management of intraoperative CO_2_ embolism. We make the following three suggestions: (a) maintenance of low CO_2_ pressure; (b) careful intraoperative EtCO_2_ monitoring; and (c) rapid management, including immediate cessation of CO_2_ insufflation, and ventilation of 100% oxygen. Medications such as epinephrine and atropine, and chest compression should be administered as needed.

Impaired swallowing and a strangling sensation are frequently reported by patients who underwent thyroidectomy. As these symptoms are subjective, they are often dismissed by clinicians. In thyroid surgery, postoperative adhesion during the healing process is associated with swallowing impairment [39]. In this study, thyroiditis and tumor size were associated with postoperative swallowing impairment, which we consider to be the result of more traction or excessive handling in cases with large tumor size or thyroiditis. Although TOETVA requires a smaller dissection area compared to other endoscopic thyroidectomy approaches, the thin flap is prone to postoperative adhesion. Our SIS-6 scores at postoperative three and six months compared favorably with other remote access thyroidectomies [40,41,42,43].

TOETVA is the best aesthetic option for thyroidectomy. It is a scarless approach with excellent cosmetic outcomes, which provides equal accessibility to either lobe without the need for an additional port. Because the specimen is extracted via the midline vestibular incision, additional skin incision on the axilla or areola is unnecessary. In the current study, after informing the patient of the risks and benefits of TOETVA, the patient selected their desired approach. We believe the selection bias favoring female patients (91.8%) is owing to the frequent cosmetics concerns of women.

For novice TOETVA surgeons, careful patient selection based on the size and location of the tumor is important. Especially for right-handed surgeons, a small nodule in the right middle or lower lobe is the best option [44]. However, TOETVA provides the midline approach to the central neck with craniocaudal view, which facilitates left lobectomy, total thyroidectomy, and CLND. In this study, 44 cases of left lobectomy and three cases of total thyroidectomy were performed without any complications. An average of three LNs were retrieved per case. Generally, TOETVA is not recommended for patients with lateral neck node metastasis or adjacent organ (i.e., trachea or esophagus) invasion. Large tumor size, and prominent mental protuberance and tubercles present technical difficulties for TOETVA. Therefore, it is recommended that in such cases, surgery is performed by an experienced surgeon [44,45]. In addition, the surgeon may not be familiar with top-down anatomy, which presents technical challenges and is time consuming, even for experienced thyroid surgeons during the initial learning curve period. Therefore, it is important for training surgeons to become familiar with top-down anatomy during open thyroidectomy, prior to attempting TOETVA.

The learning curve for a particular procedure can be defined as the number of cases required to stabilize and minimize operation time and complications. In this study, the incidence of complications was low, so the operation time was the indicator of proficiency to assess the learning curve. In the medical literature, CUSUM is frequently used to evaluate a learning curve due to its simple formulation and intuitive representation. Reaching a steady state on the CUSUM curve indicates that the learning curve has been overcome [46]. Our results indicate that the surgeon with extensive experience in open and other endoscopic thyroidectomy approaches (such as the bilateral axillo-breast approach) needed 57 cases to gain proficiency with TOETVA. This is comparable to studies reporting the learning curve for TOETVA and other endoscopic thyroidectomy approaches [47,48].

TOETVA remains an experimental surgical approach compared to the conventional open approach, which is the gold standard procedure for thyroidectomy. In this study, TOETVA was performed on patients with nodules less than 4 cm. Patients with nodules 4 cm and over were required to undergo conventional open surgery and were not included in this study. However, we expect that with advances in endoscopic technology and surgical skill acquisition, the indications for TOETVA will gradually expand. Further research on the safety and feasibility of TOETVA for larger nodules is required.

There are some limitations of this study. Because this is a single center study based on the experience of a single surgeon, it has limited generalizability for representing the surgical safety of TOETVA. In addition, the short follow-up period does not allow evaluation of long-term oncological outcomes. As TOETVA is a new technique, lack of long-term results is inevitable. Although many studies have been conducted on TOETVA, large-scale studies for malignant nodules are relatively small. Our study is meaningful because it includes more than 100 cases of malignancy. Long-term follow-up studies of oncological outcomes are needed to assess the safety and benefits of TOETVA.

## 5. Conclusions

This is a case series of our initial experience with TOETVA. As the surgeon’s experience increases, TOETVA can be performed with an acceptable operation time and low complication rate. TOETVA is a safe and feasible procedure with outstanding aesthetic and clinical results, which provides an excellent alternative for selected patients who require thyroidectomy.

## Figures and Tables

**Table 1:** Clinicopathologic characteristics of patients.

Variables	Values, *n* (%)
**Age, years** ^1^	39.7 ± 9.7
**Sex**	
Male	9 (8.2%)
Female	101 (91.8%)
**Body mass index, kg/** **m^2^** ^1^	23.5 ± 4.4
**Tumor size, cm** ^1^	1.0 ± 0.7
**Pathology**	
Malignant	101 (91.9%)
Papillary thyroid cancer	92 (83.7%)
Follicular variant papillary thyroid cancer	5 (4.6%)
Minimally invasive follicular thyroid cancer	3 (2.7%)
Minimally invasive Hurthle cell cancer	1 (0.9%)
Benign	9 (8.1%)
Follicular adenoma	4 (3.6%)
Hurthle cell adenoma	3 (2.7%)
Nodular hyperplasia	1 (0.9%)
Benign degenerating nodule	1 (0.9%)
**T stage**	
T1a	72 (71.3%)
T1b	18 (17.8%)
T2	10 (9.9%)
T3	1 (1.0%)
NA (Benign)	9
**N stage**	
Nx	14 (13.8%)
N0	64 (63.4%)
N1	23 (22.8%)
NA (Benign)	9
**Extrathyroidal extension**	
No	67 (66.3%)
Microscopic	33 (32.7%)
Gross	1 (1.0%)
NA (Benign)	9
**Combined thyroid diseases**	
No thyroiditis	81 (73.6%)
Thyroiditis	29 (26.4%)

NA, not available. ^1^ Values are expressed as mean ± standard deviation.

**Table 2:** Operative details and postoperative complications.

Variables	Values, *n* (%)
**Operative time, min** ^1^	
Lobectomy (*n* = 100)	111.0 ± 27.7
Isthmusectomy (*n* = 7)	73.7 ± 18.1
Total thyroidectomy (*n* = 3)	168.0 ± 63.4
**Central lymph node dissection**	
Performed	101 (91.8%)
Not performed	9 (8.2%)
**No. of lymph nodes** ^1,†^	
Retrieved	3.0 ± 2.5
Involved	0.5 ± 0.9
**Complications**	
Postoperative bleeding	1 (0.9%)
Surgical site infection	0 (0%)
Seroma collection	0 (0%)
Chyle leakage	0 (0%)
Vocal cord palsy	
Transient	5 (4.5%)
Permanent	1 (0.9%)
Hypoparathyroidism ^‡^	
Transient	1 (33.3%)
Permanent	0 (0%)
Mental nerve injury	0 (0%)
CO_2_ embolism	1 (0.9%)
**Open conversion**	2 (1.8%)
**Postoperative hospital stay, days** ^1^	2.4 ± 0.9

^1^ Values are expressed as mean ± standard deviation. ^†^ In 101 cases of central lymph node dissection. ^‡^ Of 3 total thyroidectomies.

**Table 3:** Clinicopathologic characteristics of patients according to groups classified by operation time in patients who underwent lobectomy for malignancy.

Variables	Short-Time Group (≤112 min)	Long-Time Group (>112 min)	*p*-Value *
**Age, years** ^1^	41.4 ± 10.6	39.0 ± 8.4	0.326 **
**Sex**			0.008 ***
Male	0 (0%)	6 (14.3%)	
Female	49 (100%)	36 (85.7%)	
**Body mass index, kg/m^2^**			0.162
<25	40 (81.6%)	29 (69.1%)	
≥25	9 (18.4%)	13 (30.9%)	
**Tumor size, cm** ^1^	0.8 ± 0.4	1.2 ± 0.8	0.012 **
**Laterality**			0.270
Rt	29 (59.2%)	20 (47.6%)	
Lt	20 (40.8%)	22 (52.4%)	
**Operation time** ^1^	91.5 ±11.6	136.2 ± 22.7	<0.001 **
**LN metastasis, n** ^1^	0.7 ± 1.9	0.4 ± 1.2	0.639 **
**Extrathyroidal extension**			0.085
No	29 (59.2%)	32 (76.2%)	
Microscopic/Gross	20 (40.8%)	10 (23.8%)	
**Thyroiditis**			0.852
No	37 (75.5%)	31 (73.8%)	
Yes	12 (24.5%)	11 (26.2%)	

LN, lymph node. ^1^ Values are expressed as mean ± standard deviation. * Chi -square test. ** Wilcoxon rank sum test. *** Fisher’s exact test.

**Table 4:** Logistic regression analysis ^1^ of risk factors associated with long-time group (>112 min).

Variables	Simple Generalized Linear Model			Multiple Generalized Linear Model		
	OR	CI	*p*-Value	OR	CI	*p*-Value
**Sex**						
Female	1.00			1.00		
Male	17.62	0.77–405.29	0.073 *	21.26	0.77–590.79	0.072
**Body mass index, kg/m^2^**						
<25	1.00			1.00		
≥25	1.99	0.75–5.28	0.166	1.55	0.50–4.84	0.451
**Laterality**						
Rt	1.00			1.00		
Lt	1.60	0.70–3.66	0.271	2.04	0.78–5.34	0.148
**Extrathyroidal extension**						
No	1.00			1.00		
Microscopic/Gross	0.45	0.18–1.12	0.088	0.54	0.19–1.54	0.252
**Thyroiditis**						
No	1.00			1.00		
Yes	1.09	0.42–2.82	0.852	1.35	0.46–3.99	0.587
**Age**	0.97	0.93–1.02	0.236	1.00	0.95–1.05	0.919
**Tumor size**	2.66	1.19–5.94	0.017	2.53	1.05–6.09	0.039
**LN metastasis**	0.88	0.66–1.18	0.403	0.89	0.62–1.28	0.526

OR, odds ratio; CI, confidence interval; LN, lymph node. ^1^ Short-time group was set as reference. * Logistic regression with Firth’s method.

## Data Availability

The data presented in this study are available on request from the corresponding author.
